# Endoscopic closure of a large gastric mucosal defect using a novel endoscopic suturing device in a porcine model

**DOI:** 10.1055/a-2897-7502

**Published:** 2026-06-30

**Authors:** Qiong Yang, Zhanhui Ye, Xiujie Ye, Siqi Wang, Kequan Chen

**Affiliations:** 1 Department of GastroenterologyThe First Affiliated Hospital of Guangzhou Medical UniversityGuangzhouGuangdongChina

**Fig. 1:**
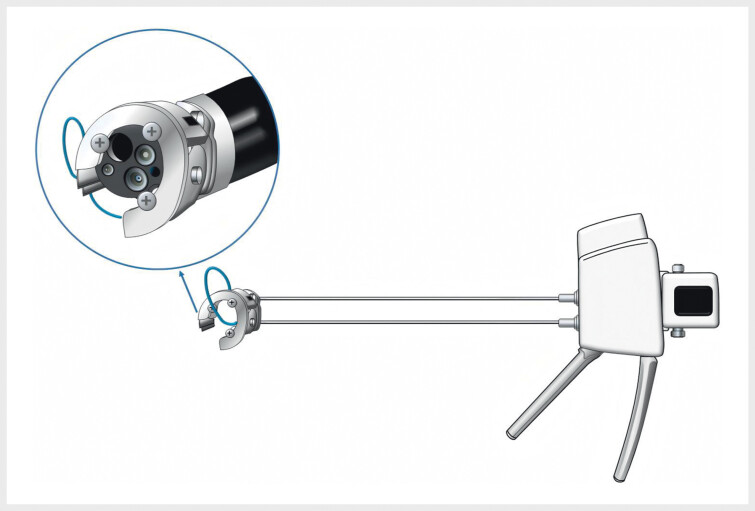
Schematic view of the novel endoscopic suturing device. The device comprises a control handle, transmission components, and a suture-loaded advancing assembly with a distal C-shaped opening and a handle-controlled suturing needle (fabricated by DERUI MEDICAL, Guangxi, China).

Management of large mucosal defects after endoscopic submucosal dissection (ESD) remains important in endoscopy. Technical complexity, limited accessibility, and cost constrain current closure platforms. 1​ Although endoscopic hand suturing enables needle-and-suture closure, it requires specialized accessories and advanced expertise. 2​ Here, we present a novel endoscopic suturing device for closure of large gastric defects ( Fig. 1 ). The device is compatible with gastroscopes with a distal outer diameter of 9.9 mm and colonoscopes with a distal outer diameter of 11.7 mm, both with a 3.2-mm working channel. Its feasibility was evaluated in a live porcine model with a large gastric defect created by ESD.

**Fig. 2:**
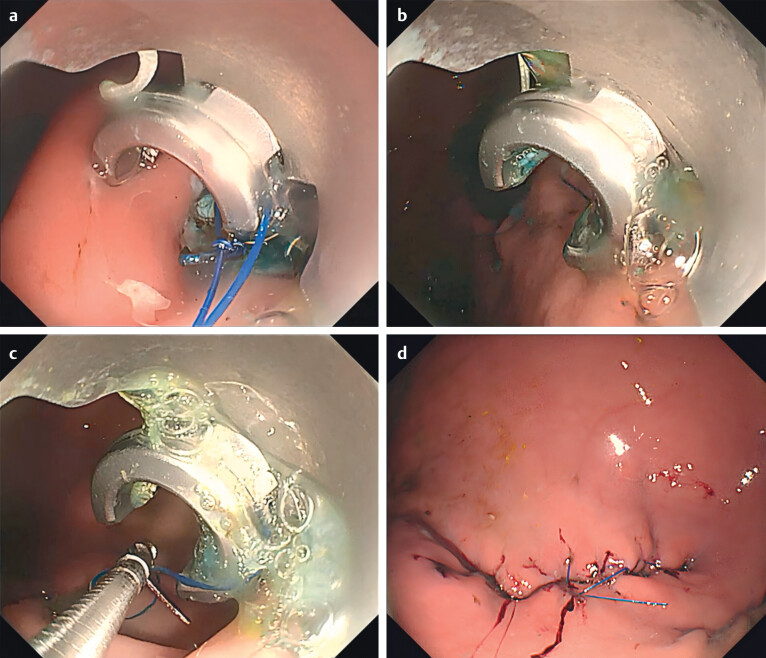
Procedure for closing a gastric defect using the novel endoscopic suturing device. ( **a** ) The distal C-shaped opening was aligned with the wound edge, and suturing was performed by manipulating the handle-controlled needle. ( **b** ) Gentle withdrawal of the endoscope tightened the suture and improved edge apposition. ( **c** ) The excess suture was cut with scissors after suturing. ( **d** ) Completed closure of the defect.

Suturing was performed from proximal to distal as follows ( Video 1 ). The distal C-shaped opening was aligned with the wound edge, and suturing was performed by controlling the handle ( Fig. 2a ). After the first stitch, the endoscope was manipulated to release the knot, and the first knot was secured by tightening the suture. Continuous suturing was repeated along the defect, with gentle withdrawal of the endoscope after every two or three stitches to tighten the suture and improve apposition ( Fig. 2b ). Suturing was completed with 12 stitches. The excess suture was cut with scissors ( Fig. 2c ), and closure was completed ( Fig. 2d ).

**Video 1** Endoscopic closure of a large gastric mucosal defect using a novel endoscopic suturing device.


**Fig. 3:**
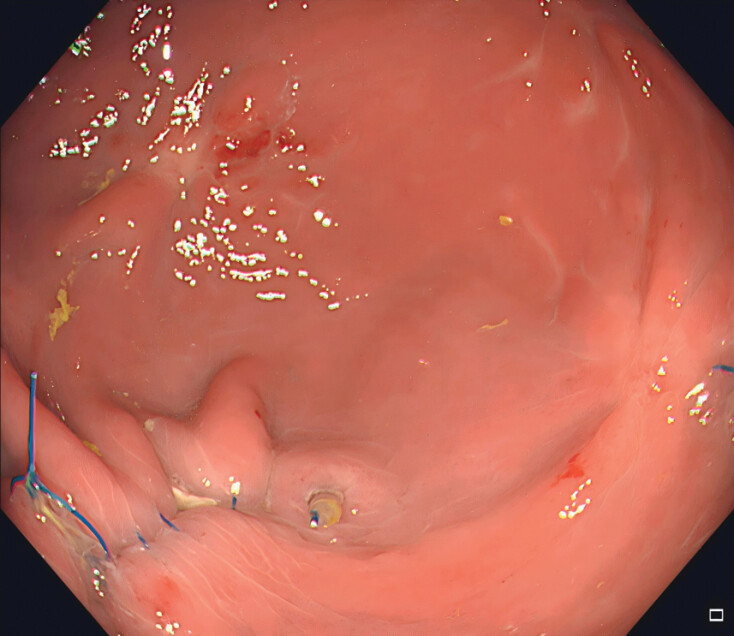
Endoscopic follow-up at 1 week after suturing showed close apposition of the defect without complications.

Endoscopic reassessment at 1 week showed persistent close apposition of the sutured defect ( Fig. 3 ), without tissue tearing, knot slippage, delayed bleeding, suture loss, or other adverse events. Compared with established hand-suturing, clip-based, or tack-based methods, 2​ 3​ ​4​ this device combines tissue capture through a distal C-shaped opening with handle-controlled, needle-mediated repeated suturing under direct endoscopic visualization, potentially facilitating secure approximation of large gastric or full-thickness gastrointestinal defects. Given that endoscopic suturing has been used in procedures related to endoscopic sleeve gastroplasty, 5​ this device may be explored for gastric remodeling.

Endoscopy_UCTN_Code_TTT_1AO_2AO
